# Urgent need for a randomized controlled trial with only septic patients!

**DOI:** 10.1186/s13613-019-0596-6

**Published:** 2019-10-21

**Authors:** Sébastien Redant, Matthieu Legrand, Yael Langman, Alejandra Garcia Aguilar, Keitiane Kaefer, David De Bels, Rachid Attou, Kianoush Kashani, Patrick M. Honore

**Affiliations:** 1 ICU Department, Centre Hospitalier Universitaire Brugmann-Brugmann University Hospital, 4, Place Arthur Van Gehuchten, 1020 Brussels, Belgium; 2 Department of Anesthesiology, Critical Care and Burn Unit, St. Louis Hospital, UMR-S942, Inserm, University Paris 7 Denis Diderot, Paris, France; 3 Hospital General de Zona N°14 IMSS, Universidad Autónoma de Guadalajara, Zapopan, Mexico; 4 Division of Nephrology and Hypertension, Division of Pulmonary and Critical Care Medicine, Mayo Clinic, Rochester, USA

We have read with great interest the meta-analysis of Xue et al. on the effects of chloride-rich crystalloid solutions in critically ill patients [1]. While the authors found no significant difference in survival, the severity of acute kidney injury (AKI), and the need for renal replacement therapy (RRT), which they noted in septic patients with hyperchloremic acidosis,the use of normal saline was associated with increased mortality [1]. These findings have also been previously reported [2]. High chloride level in the macula densa would activate the glomerulo-tubular feedback causing vasoconstriction of the afferent arteriole. In addition, chloride facilitates thromboxane release, which leads to vasoconstriction. Hyperchloremia also enhances the effect of angiotensin II receptor blockers [3]. In one study, increase in chloride level > 5 meq/L was found to be an independent risk for AKI [4]. Negative effects of induced acidosis, lack of signal in previous large randomized controlled trials (RCTs), and potential consequences of hypo-osmolality of balanced solutions add to the arguments on benefits and disadvantages of chloride-rich solutions [3, 4]. In sepsis, using large volume of chloride-rich fluids would lead to hyperchloremic acidosis.The impact of hyperchloremia among those with other reasons of afferent arteriolar vasoconstriction is more obvious. Hence, the likelihood that a randomized controlled trial (RCT) showing the impact of chloride-rich solutions on outcomes in patients with higher severity of illness is substantially higher. Cost-effectiveness of the use of chloride-rich solutions should be investigated as well [3, 4]. New statistical approaches like the trial sequential analysis (TSA) together with a meta-analysis could add to the current literature. Indeed, TSA might help in justifying needs for further RCTs as suggested by Xue et al. [1]. TSA might also help defining the minimal number of patients that needs to be included in order to have a well powered (next) RCT [1]. Thongprayoon et al. reported that the impact of high chloride level on mortality is significantly higher in the presence of anion gap metabolic acidosis, and as septic patients have higher lactate, it makes them an appropriate target population [5]. As chances of worse clinical outcomes in response to chloride-rich solutions are even higher in septic patients, we suggest in the next RCT that instead of focusing on all critically ill patients, only patients with sepsis and septic shock requiring fluid resuscitation are to be considered as they require larger volume and already have a higher risk of poor outcomes. It is important to assess the non-inferiority of normal saline in comparison with balanced solutions among septic patients with high risk of AKI, need for RRT, and death. Indeed as suggested by the authors, subgroup analysis and TSA revealed trends toward cumulative evidence that patients without traumatic brain injury (TBI) (in other words septic patients) would benefit from the use of balanced crystalloid fluid. Previous studies showed 0.9% saline solutions could benefit TBI patients by reducing complications of cerebral edema and intracranial hypertension.

## Data Availability

Not applicable.

## Abbreviations

AKI: acute kidney injury
RRT: renal replacement therapy
RCTs: randomized controlled trial
TSA: trial sequential analysis
TBI: trauma brain injury
